# Hematologic parameters and Neutrophil / Lymphocyte ratio in the prediction of urethroplasty success

**DOI:** 10.1590/S1677-5538.IBJU.2018.0682

**Published:** 2019-04-01

**Authors:** Ramazan Topaktaş, Ahmet Ürkmez, Emre Tokuç, Mehmet Akyüz, Musab A. Kutluhan

**Affiliations:** 1 Department of Urology, Haydarpaşa Numune Training and Research Hospital, Istanbul, Turkey

**Keywords:** Inflammation, Neutrophils, Urethral Stricture

## Abstract

**Objective:**

The pathophysiology of urethral stricture and its recurrence remains vague and one of the important causes is progressive inflammation. It has been shown in recent years that the neutrophil / lymphocyte ratio is a marker of systemic inflammation and is associated with prognosis in many cardiovascular diseases, malignancies and chronic inflammatory diseases. We assessed simple systemic inflammation markers preoperatively and surgical techniques for urethral stricture recurrence after urethroplasty.

**Patients and Methods:**

After exclusion criteria applied, a total of 117 male cases operated with urethroplasty in our clinic between January 2012 and June 2017 were included in the study and analyzed retrospectively. Localization and length of the strictures of the patients, neutrophil counts and percentages, lymphocyte counts and percentages, and neutrophil / lymphocyte ratios in preoperative peripheral blood samples were statistically analyzed. Recurrent stricture during first 12 months follow-up after the surgery has been assessed as recurrence.

**Results:**

The mean age of the patients was 54.12 ± 16.35 and the mean urethral stricture length was 3.44 ± 1.83 cm. Recurrence was observed in 30.1% of cases who received buccal graft, 30% in penile skin applied cases and 26.1% of cases treated with end-to-end anastomosis and there was no statistically significant difference between neutrophil, lymphocyte, neutrophil / lymphocyte ratio and average stricture segment length between recurrent and non-recurrent cases (p > 0.005).

**Conclusions:**

We consider that neutrophil, lymphocyte counts and their ratio prior to urethroplasty and the technique performed are not parameters that can be used to predict stricture recurrence. Prospective and randomized new trials with larger patient populations are needed to make more accurate judgments about the role of these inflammatory parameters.

## INTRODUCTION

Urethral stricture is a pathology caused by fibrosis and stenosis of the urethral epithelium and corpus spongiousum, leading to lower urinary tract symptoms and therefore, it is a troublesome problem in urology for both the patient and the physician. It can significantly disrupt quality of life and can cause serious amounts of health expenditure. At present, the incidence of urethral stricture may be iatrogenic due to increased application of transurethral procedures in urology practice, as well as trauma, urethral catheterization, and urinary infections (1).

The treatment of the urethral stricture varies according to the location, length, and type of stenosis, and endoscopic methods are generally preferred because they are simpler, more economical, and repeatable (2). According to the guidelines, urethroplasty is recommended when the segment of the stenosis is 2 cm or more, endoscopic methods have failed, there is excessive periurethral fibrous tissue density, or a high risk of recurrence (3). The natural course of the urethral stricture is blurred, but the well-known feature is its repetitive character. For this reason, efforts to investigate different markers and treatment alternatives in order to predict and prevent recurrence are on the rise.

The neutrophil / lymphocyte ratio (NLR) can be used as a potential predictive marker to detect inflammation, which is easily calculated through blood count analysis (4). The NLR may help foresee recurrence risks after urethroplasty because inflammation is thought to have a major role in the pathogenesis of urethral stricture.

According to the literature, there are inconsistencies about the predisposing factors regarding recurrent strictures after urethroplasty, and this issue is not clear (5). To our knowledge, no other study has used systemic inflammation markers to predict recurrence after urethroplasty procedures performed due to urethral stricture. In this study, we evaluated preoperative inflammation markers in relation to the recurrence of urethral stricture after urethroplasty and surgical techniques.

## MATERIALS AND METHODS

A total of 117 male patients who underwent urethroplasty due to urethral stricture in our clinic between January 2012 and January 2017 were retrospectively reviewed from the hospital electronic database after receiving written informed consent from the patients. Approval was obtained from the local ethics committee (No: 2017 / 87-15). All patients had undergone urethroplasty due to urethral stricture greater than 2 cm in various locations of the urethra. Patients with malignancies; diabetes mellitus; cardiac, pulmonary, hematologic, liver or kidney dysfunction; patients who received blood transfusions; patients with a body mass index (BMI) over 35 kg / m^2^; and those with a history of previous open urethral surgery were excluded. The location and length of the stricture were calculated using retrograde urethrography and cystoscopy. Urine culture tests were performed on each patient preoperatively and the surgeries of patients with active infections were postponed until the treatment was completed. Neutrophil and lymphocyte counts were analyzed the day before the operation and the NLR was calculated by dividing the number of neutrophils by the number of lymphocytes. Blood counts of all patients were made on a stationary device in the hospital's central laboratory that is regularly checked. The location and length of the stricture, neutrophil numbers and percentages, lymphocyte counts and percentages, and NLRs in preoperative peripheral blood samples were examined in detail and statistically analyzed. All procedures were performed by a single experienced surgeon. After receiving an appropriate prophylactic single dose of antibiotic, all operations were performed in lithotomy position. In all patients it was inserted a 16-F or 18-F silicone catheter after the operation. When needed, paracetamol (1000 mg) which has less peripheral and anti-inflammatory effects, was administered intravenously once a day in postoperative period. The success of urethroplasty was evaluated through urine flow rate (maximum flow rate > 10 mL / sec) or no recurrent stricture in retrograde urethrography within 12 months after surgery.

### Statistical analysis

Statistical analyses were performed using the Statistical Package for the Social Sciences for Windows version 18.0 (SPSS, Chicago, IL, USA). Expression ratios were transformed into fold changes and reported as relative expression. The normal distribution of the parameters was evaluated using the Shapiro-Wilks test. Descriptive statistical methods (mean, standard deviation, frequency) were used, and Student's t-test was used to compare quantitative parameters between two groups that were distributed normally. The Chi-square test was used for the comparison of qualitative data. P < 0.05 was regarded as significant.

## RESULTS

The mean age of 117 male patients included in our study was 54.12 ± 16.35 (range, 14 to 84) years, and the mean urethral stricture length was found as 3.44 ± 1.83 cm. Totally, 68 patients had only bulbar urethral stricture. Other urethral stricture locations and percentages are detailed in Table-1. Most patients presented with obstructive symptoms (79.2%), while acute urine retention and suprapubic catheter fixation were the presenting symptoms in 8.7%.

**Table 1:** Stricture location and surgical method parameters.

		n	%
**Location**			
	Bulbar and membranous urethra	3	2.6
	Bulbar urethra	68	58.1
	Distal bulbar urethra	8	6.8
	Distal and proximal bulbar urethra	1	0.9
	Fossa navicularis	3	2.6
	Fossa navicularis and bulbar urethra	2	1.7
	Fossa navicularis and penile urethra	1	0.9
	Membranous urethra	2	1.7
	Distal and proximal penile urethra	1	0.9
	Penile urethra	15	12.8
	Penile urethra and distal bulbar urethra	4	3.4
	Proximal bulbar urethra	8	6.8
	Proximal penile urethra	1	0.9
	Method		
	Buccal Graft	73	62.4
**Bulbar**		1	0.9
	Penile skin Flap	20	17.1
	End-To-End Anastomosis	23	19.7

Regarding the techniques used among open urethroplasty operations, the buccal graft method was used in 62.4% of cases, end-to-end anastomosis in 19.7%, penile skin flap method in 17.1%, and the bulbar method was used in 0.9% (Table-1). The mean operative duration was 132.5 ± 16.35 minutes. The mean withdrawal time of the urethral catheter was 15 (range, 12 to 21) days. A total of 34 patients (29%) had recurrence. There were no statistically significant differences between the recurrence rates according to the urethroplasty method used (p > 0.05). Recurrence was seen in 30.1% of cases where the buccal substitution method was used, 30% of cases with penile skin, and 26.1% of cases with end-to-end anastomosis. These results are shown in Table-2.

**Table 2:** Recurrence evaluation according to surgical method and blood parameters.

	Recurrence		
**Method**	**Yes**	**No**	**p** *
	**n (%)**	**n (%)**	
**Buccal graft**	22 (30.1)	52 (69.9)	0.931
**Penile skin flap**	6 (30.0)	14 (70.0)	
End-To-End anastomosis	**6 (26.1)**	**17 (73.9)**	
	Recurrence		
	**Yes**	**No**	**p** **
	**Mean±SD**	**Mean±SD** ***	
**Neutrophil**	4.9±1.66	4.98±1.81	0.824
**Lymphocyte**	2.31±0.87	2.23±0.91	0.649
**Neutrophil/Lymphocyte**	2.36±1.06	2.58±1.49	0.418
**Stricture length (cm)**	3.41±1.76	3.46±1.97	0.907

* p value of Chi-square test

** p value of Student- t test

*** Standard deviation

There were no statistically significant differences between neutrophils, lymphocytes, NLRs, and mean stricture lengths between recurrent and non-recurrent cases (p > 0.005) (Table-2).

## DISCUSSION

Although urethral stricture has been a well-known disease for many years, urologists still strive to find a way to prevent recurrence or a curative method. Often seen in males, urethral stricture has an incidence of 0.2-1.2%, which increases over the age of 55 years (6). Anatomically, the urethra is divided into two parts: anterior and posterior. The anterior part, wrapped by corpus spongiosum, comprises two parts: penile and bulbar. The bulbar urethra lies between the penoscrotal junction and the membranous urethra. The posterior urethra is located between the bladder neck and the bulbar urethra and it comprises the prostatic urethra and the membranous urethra covered with the external urethral sphincter (7).

Penile urethral strictures can often be due to urinary intervention, urethral infection or inflammation, or can occur in older men due to ischemia. Bulbar urethral strictures are the most common site of urethral stricture, and most of these strictures are congenital or idiopathic (8). When urethral strictures are considered, fibroblasts are probably responsible for the development of the urethral stricture; however, the reason for the urethral stricture is related to the urinary extravasation into the subepithelial space causing increased inflammation and subsequent scar formation. With this knowledge, many authors have used colchicine, mitomycin-C, triamcinolone, corticosteroids, and anti-inflammatory drugs locally or systemically to reduce urethral stricture after urethral procedures (9). In this context, urethral stricture is a result of inflammatory changes in the epithelium of the urethra and can be treated by interfering with the inflammatory process. We used anti-inflammatory drugs (COX-2 inhibitors) three days after the operation to reduce inflammation in our cases.

The treatment choice after urethral stricture is related to the stricture length, location, and recurrence. In most urethral strictures, direct visual internal urethrotomy (DVIU) or urethral dilatation is preferred as a starting treatment. In recurrent urethral strictures, DVIU is thought to cause extra scar formation, and thus the length and severity of stenosis are affected negatively. For this reason, open urethroplasty in patients with recurrent urethral strictures, long urethral stricture, or dense periurethral fibrous tissue has a higher success rate and less chance of recurrence than DVIU. The gold standard treatment for short and apparent strictures of the bulbar urethra, whether or not the lumen is completely obliterated, is excision, spatulation of both ends, and end-to-end anastomosis (10). For bulbar urethral strictures equal or longer than 2 cm, the gold standard method is stricturotomy and dorsal patch substitution urethroplasty with a buccal mucosal graft (11). Urethroplasty using carefully-prepared penile shaft skin with a rich dartos pedicle can be performed in patients with a long bulbar or penile stricture completely obstructed with extreme spongiofibrosis, even if it is not a gold standard method (12). We chose to perform open urethroplasty instead of internal urethrotomy in strictures over 2 cm and with dense fibrosis.

The reason for recurrent urethral strictures after urethroplasty remains unknown. However, it is known that there may be a relationship between past urinary procedures, patient age, mucosal damage, inflammation, and systemic diseases with urethral stricture (13). In addition, according to the literature, stricture recurrence risk is likely to increase as the length of the urethral stricture segment increases (14). This is due to the increased surface area required for a successful graft placement as the stricture length increases. This leads to ischemic strictures being seen often (15). There was no significant relationship between urethral stricture segment and urethral stricture recurrence in our study. We think that this is caused by the fact that our study did not include a wide range of groups in terms of urethral stricture length. In addition, despite evaluating factors that might cause urethral strictures in our study, no significant relationship between the development of urethral strictures after urethroplasty and preoperative neutrophil count, lymphocyte count, NLR, stricture location / length, and surgical technique was found.

In recent years, it has been shown that the NLR may be a marker of chronic systemic inflammation and is associated with prognosis in many cardiovascular diseases, malignancies, and chronic inflammatory diseases. These markers were previously assessed in various uro-oncologic cases and have been shown to have an effective role in both predicting postoperative surgical margin status and progression-free survival (16, 17). It is known that white blood cells differ in systemic inflammation, such as neutrophilia and lymphopenia (18). This inflammatory response and tissue necrosis leads to fibrosis and poor recipient vascularity, which likely has a key role in deficient wound healing, which threatens urethroplasty success (19). In a study that regarded an NLR of 2.7 as a limit, it was shown that a combination of tumor stage and NLR could be used to assess the risk of recurrence in patients with non-metastatic renal cell carcinoma (20). Another study showed that the NLR in non-clear-cell kidney tumors was an independent prognostic factor for disease-free survival after curative surgery. As such, the NLR has been reported to be a significant marker for patient counseling and clinical trial design (21). In a related study of 208 patients with a history of urethral stricture after transurethral resection of the prostate from our country, it was shown that the NLR was relatively higher in relapsed patients but not significant (9). In our study, there were no statistically significant differences between neutrophils, lymphocytes, NLR, and patients with and without stricture recurrence after urethroplasty.

A correlation between coronary artery disease, diabetes mellitus, morbid obesity, peripheral vascular disease, and chronic obstructive pulmonary disease and urethroplasty failure has been shown in previous studies (22, 23). Here, pathophysiologic components such as decreased tissue vascularity, chronic inflammation, impaired collagen synthesis, and ischemic strictures increase urethroplasty failure.

It is conceivable that there may be a correlation between increased age and recurrence of stenosis because the comorbid diseases known to be closely related to urethroplasty failure / urethral stricture recurrence are more frequent in older patients. However, when the literature is examined, no correlation has been reported between age and urethroplasty failure (24). In our study, patients with systemic diseases that could adversely affect wound healing were excluded from the study, so the average age was not high.

The main limitations of our study are that it is single-centered, it has a relatively limited number of patients, its retrospective nature, and the exclusion of older patients due to additional comorbidities. Therefore, patients with these comorbid factors and patients with a body mass index above 35 kg / m^2^ were not included in our study.

## CONCLUSIONS

Neutrophil and lymphocyte count and the NLR are inexpensive, easily accessible laboratory tests that can be calculated with a simple blood count. Although it is not yet possible to say that the consideration to the surgical technique to be applied along with neutrophil, lymphocyte numbers and the NLR before urethroplasty was successful in predicting the recurrence of stenosis, we think that the presence of a systemic inflammatory response may be an important marker, as it is in oncologic cases. We believe that these laboratory findings should be supported by large series, prospective, comparative, randomized, and multi-centered studies with long-term results to detect the association of urethral stricture recurrence after urethroplasty, and to obtain stronger judgments.
